# Analysis of Flaw Detection Sensitivity of Phased Array Ultrasonics in Austenitic Steel Welds According to Inspection Conditions

**DOI:** 10.3390/s21010242

**Published:** 2021-01-01

**Authors:** YoungLae Kim, Sungjong Cho, Ik Keun Park

**Affiliations:** 1 Graduate School, Seoul National University of Science & Technology, Seoul 01811, Korea; kyl394@seoultech.ac.kr; 2 NDT Research Center, Seoul National University of Science and Technology, Seoul 01811, Korea; cho-sungjong@seoultech.ac.kr; 3 Department of Mechanical and Automotive Engineering, Seoul National University of Science and Technology, Seoul 01811, Korea

**Keywords:** phased array ultrasonic testing (PAUT), austenitic steel welds, anisotropic, flaw detection sensitivity, inspection conditions

## Abstract

The anisotropy and inhomogeneity exhibited by austenitic steel in welds poses a challenge to nondestructive testing employing ultrasonic waves, which is predominantly utilized for the inspection of welds in power plants. In this study, we assess the reliability of phased array ultrasonic testing (PAUT) by analyzing the flaw detection sensitivity of ultrasonic beams in anisotropic welds, based on the inspection conditions. First, we simulated the sectorial scan technique, frequently employed for the inspection of actual welds, while taking into account the ultrasonic wave mode, frequency, and shape and position of a flaw. Subsequently, we analyzed the flaw sensitivity by comparing A-scan signals and S-scan results. The sensitivity analysis results confirmed the detection of all flaws by considering at least two inspection methods based on the shape and position of the flaw. Furthermore, we verified our model by performing an experiment under the same conditions as the simulation and found that the results were in agreement. Hence, we find that the simulation modeling technique proposed in this study can be utilized to develop suitable inspection conditions, according to the flaw characteristics or inspection environment.

## 1. Introduction

To ensure the long-term safe and reliable operation of nuclear and thermal power plants, it is important to secure their structural integrity by nondestructive testing (NDT). NDT is applied to the in-service inspection (ISI) and pre-service inspection (PSI) of power plants and mainly involves radiography testing (RT) [1,2]. In the case of RT, there are safety issues, such as radiation exposure. Accordingly, it is possible to perform volumetric examination, and it is being replaced by ultrasonic examination that is harmless to the human body. Among various ultrasonic testing techniques, phased array ultrasonic testing (PAUT) determines the angle and focal law and transmits pulses through the electromagnetic time delays of multiple elements arrayed in phased array (PA) probes to generate a focused beam. It can detect flaws present in multiple directions of the target under inspection. PAUT can obtain the images of the inside of a specimen in real time through linear/electronic scan, enabling high-speed electronic scanning within the length range of the PA probe, as well as sectorial scan (S-scan), which can create the required sectorial beam using a PA probe [3,4,5]. Consequently, this technique has been applied in various areas, such as power plants [6,7,8,9], railways [10,11,12], aerospace [13,14,15], and construction [16,17]. In particular, power plants constitute a significant application field of PAUT, which is applied in ISI for the welded joints of power plant containers or other facilities. 

Austenitic steel, exhibiting excellent acid and corrosion resistance, is commonly used in power plants. However, austenitic steel welds exhibit anisotropy and inhomogeneity, which are caused by the solidification of the metal, and are related to grain orientation. The accurate description of actual welds is difficult due to the complicated microstructure, and they are known to have coarse grains, which have unevenly distributed directions and sizes [18,19,20,21]. The crystal structures of these metals decrease the propagation of ultrasonic waves inside the materials due to phenomena such as refraction, scattering, and attenuation, caused by the distortion of ultrasonic waves at the grain boundaries. Furthermore, the time delays in PAUT require information on nominal specifications (properties and geometry of the material), and they are calculated based on the assumption that the material exhibits isotropy and homogeneity. Therefore, the beam focusing performance deteriorates and the signal-to-noise ratio (SNR) decreases for welds that exhibit anisotropy and inhomogeneity. Consequently, it is difficult to detect flaws in welds and evaluate their reliability using PAUT. To complement this, numerous studies have been conducted on PAUT, including automatic beam focusing technology [22,23] and the total focusing method (TFM) [24,25,26]. Most studies have focused on measurement technology, signal processing techniques, and sensor development; these require time for field application, as they are still being conducted in laboratories. In most fields, the material is assumed to be isotropic and homogeneous, as described above, and scan plans are prepared under such conditions. Therefore, reliable and quantitative evaluations have not been performed properly. For highly reliable weld inspection at the current PAUT technology level, it is necessary to understand the propagation characteristics of phased array ultrasonic beams in welds and develop a procedure considering the flaw types and inspection conditions. 

In this study, we analyze the propagation characteristics of PA ultrasonic beams in welds through simulation, taking into account the inspection conditions. In the simulation, the actual boiler tube (SUS304H) and PA probe used in the experiment were modeled and used to reflect the actual inspection conditions as accurately as possible. Appropriate inspection conditions were determined through the simulation results, and the flaw detection sensitivity was compared and analyzed according to the flaw type. An experiment was carried out in the actual boiler tube under the same inspection conditions as in the simulation, confirming the accuracy of the simulation modeling technique used in this study as well as the inspection conditions developed by the simulation.

## 2. Austenitic Steel Weld and PA Probe Modeling

### 2.1. Austenitic Steel Weld Modeling

**Figure 1:**
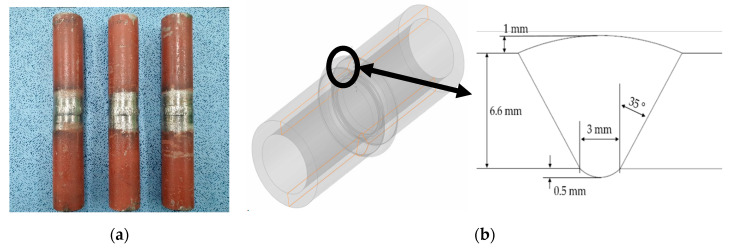
Austenitic steel tube (SUS304H) used in thermal power plant boilers and their modeling; (**a**) specimen, (**b**) weld geometry of tube.

**Figure 2:**
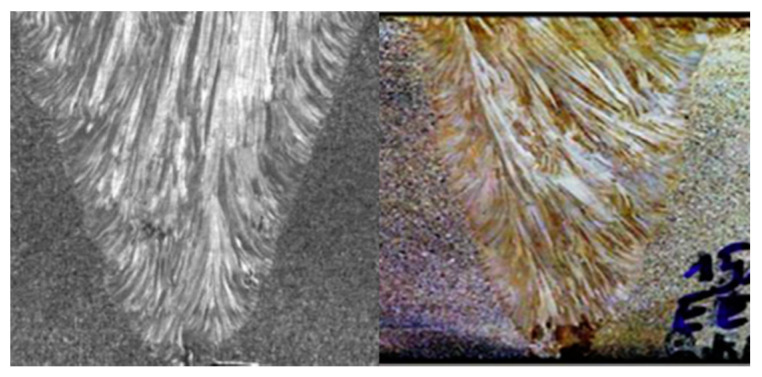
Metal crystal structure of the austenitic steel weld [27,28].

To reflect the actual inspection conditions as accurately as possible, we modeled a tube (SUS304H) that is used in thermal power plant boilers. Figure 1a,b show the actual tube and the geometry and dimensions of the modeled tube, respectively. The tube was welded using the same metal, and had an overall diameter of 42.2 mm and thickness of 6.6 mm. The specifications of the weld included a 35° bevel angle, 1 mm top bead, 0.5 mm root bead, and 3 mm root gap width. The material properties of the tube base metal used for the simulation were $\rho =7800\;\mathrm{kg}/m^{3}$, $V_{L}=5900\;m/s$, and $V_{s}=3230\;m/s.$ Subsequently, weld modeling was performed to show the anisotropy and inhomogeneity of the weld. Figure 2 shows the metal structure of the weld. The size and direction of crystal grains are determined according to various parameters, such as the welding temperature and cooling rate [27,28]. Although the grain orientation slightly differs during solidification depending on various conditions, the grains usually grow vertically on the groove surface, and their orientation slightly changes toward the center.

**Figure 3:**
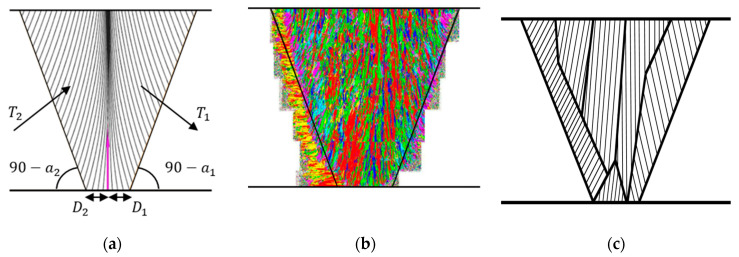
Weld modeling method. (**a**) Ogilvy’s model, (**b**) classification into domains with electron backscatter diffraction (EBSD) scanning [31], (**c**) classification into six domains with uniform orientation.

The Ogilvy model is a representative method for analyzing the anisotropic crystal structure of welds [29,30]. Figure 3a shows the crystal structure of the Ogilvy model, which can be expressed as shown in Equation (1).

(1) $$F_{1}\left(y,z\right)=tan\theta_{1}=\;\frac{-T_{1}\left(D_{1}+ztan\alpha_{1}\right)}{y^{\eta }},\;y\geq 0\;F_{2}\left(y,z\right)=tan\theta_{2}=\;\frac{T_{2}\left(D_{2}+ztan\alpha_{2}\right)}{{\left(-y\right)}^{\eta }},\;y<0\;,$$

where $F_{1}$ and $F_{2}$ represent the columnar grain of the weld and are divided into negative and positive regions based on the center of the weld. θ is the grain orientation for the positive and negative domains, based on the center of the weld, and *T* is proportional to the grain slope. *D* denotes half the root gap, and *α* is the angle between the center of the weld and the side wall. η is a variable from 0 to 1, which measures the change in grain orientation as a function of y. Because the Ogilvy model exhibits an ideal symmetrical structure, as shown in Figure 3a, it is difficult for it to demonstrate the asymmetrical inhomogeneity of the actual anisotropic weld, as shown in Figure 2. Figure 3b shows a modeling method by classification into domains with uniform grain orientation after examining the crystalline structure matrix of the weld using Macro-Photo and EBSD scanning [31]. This method reflects the metal structure of the actual weld as accurately as possible; however, it is less practical, because the analysis time increases significantly with the number of domains. 

To understand the metal crystal structure of the weld and further assess Figure 3a,b, the weld was modeled as shown in Figure 3c. The grain orientation was set as vertical on the side wall and slightly changed toward the center, as in the Ogilvy model, to reflect the characteristics of the metal crystal structure of the weld. The properties of a material vary depending on the orientation of the metal crystal structure, which is referred to as the anisotropy. To determine the anisotropy of the material, the modulus of elasticity related to the mechanical structural strength was used, as shown in Equation (2) [32].

(2) $$K=\left(\begin{array}{cccccc} 216.0 & 145.0 & 145.0 & 0 & 0 & 0\\ & 262.75 & 98.25 & 0 & 0 & 0\\ & & 262.75 & 0 & 0 & 0\\ & & & 82.25 & 0 & 0\\ & sym & & & 129.0 & 0\\ & & & & & 129.0 \end{array}\right)\mathrm{GPa}$$

The modulus of elasticity ($\theta =0^{{}^{\circ}}$) in Equation (2) was applied after setting the angle (θ) for the *x*–*z* plane and changing it according to the grain direction [32,33,34]. 

Furthermore, modeling was performed by classification into six domains with uniform grain orientation by referring to the metal crystal structure of the weld in Figure 2 to achieve asymmetry. Here, the simulation was performed while the number of domains was increased from 3 to 24 using the CIVA software, a nondestructive platform from EXTENE. Table 1 summarizes the analysis time, according to the number of domains. The Ogilvy model required approximately 30 min, and the model proposed in this study required 30 min when the number of domains was 3, and 300 min when it was 24. Despite the short analysis time, three domains could not effectively reflect the characteristics of the weld. Thus, six domains were used in this study, as their analysis time was similar to that of the Ogilvy model, and they were expected to effectively reflect the characteristics of the weld based on the simulation results.

### 2.2. Probe Modeling

**Figure 4:**
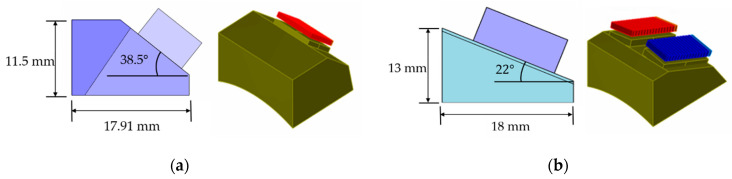
Phased array (PA) probe modeling for simulation; (**a**) longitudinal 2D linear, (**b**) transverse.

In this study, the height of the flaw was set to 1 mm. It is known that, when the particles, such as rolled steel, are fine and the SNR is high, the size of defects that can be detected by ultrasonic inspection is about half the wavelength (λ). The appropriate frequency was selected in the consideration of the commercial probe and wavelength, that is mainly used in the field. In this scenario, the frequencies and wavelengths used in the experiments and simulation were a 5 MHz (λ: 1.18 mm) longitudinal wave and 4 MHz (λ: 0.81 mm) and 7.5 MHz (λ: 0.43 mm) transverse waves. Figure 4 shows the PAUT probe modeled for the simulation. The elements are arrayed in two rows in the case of longitudinal waves. The use of the 2D linear array probe, which separates transmission and reception (TR), can achieve relatively good results in austenitic steel structures, as it eliminates the dead zone and improves the SNR [34]. In the case of longitudinal waves, mode-converted signals are generated when reflection occurs from the flaws or bottom surface, making signal analysis difficult. Therefore, transverse waves, which have a relatively straightforward signal analysis, have been frequently used. In the case of transverse waves, a high frequency is generally used to increase the resolution, whereas a low frequency is used to increase the transmittance for specimens with severe attenuation or scattering.

These requirements were reflected, and the probes used in the actual PAUT were selected by varying the probe geometry, center frequency, and wave mode (longitudinal/transverse), as shown in Table 2. Olympus products were used for the selected probes and wedges, 12X5-A2 combination was used for the longitudinal wave, and a CCEV-A15 combination was used for the transverse wave.

## 3. Analysis of Propagation Behavior of PA Ultrasonic Beams

**Figure 5:**
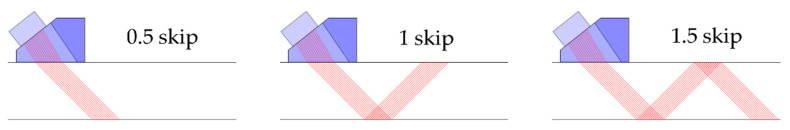
Beam propagation according to skip distance.

We performed a simulation to analyze the propagation behavior of PA ultrasonic beams in welds. The beam propagation behavior simulation is a method that visually confirms refraction, attenuation, and scattering, while the ultrasonic beam propagates as a result of combining all propagation behaviors for each angle in the set scanning range. The simulation was conducted for longitudinal and transverse waves, and the specimens and probe used were the same as those in Section 2. In the case of the longitudinal waves, up to 0.5 skip distance and a 5 MHz frequency were employed to simplify the analysis, which was difficult due to mode-converted signals. In the case of the transverse waves, up to 1 skip distance was assumed, and the simulation was performed at frequencies of 4 and 7.5 MHz to compare the transmittance of the beam. The skip distance refers to the distance that the ultrasonic beam reflects off the bottom surface, and Figure 5 shows the beam propagation according to skip distance. The S-scan, which is used predominantly in the field, was employed as the scanning method in the simulation. The scanning angle used 40°–75° of the transverse wave and 55°–85° of the longitudinal wave to cover the inspection volume. To analyze the propagation characteristics in anisotropic welds, the simulation was also performed in isotropic welds, and the results were compared.

### 3.1. Beam Computation Simulation

**Figure 6:**
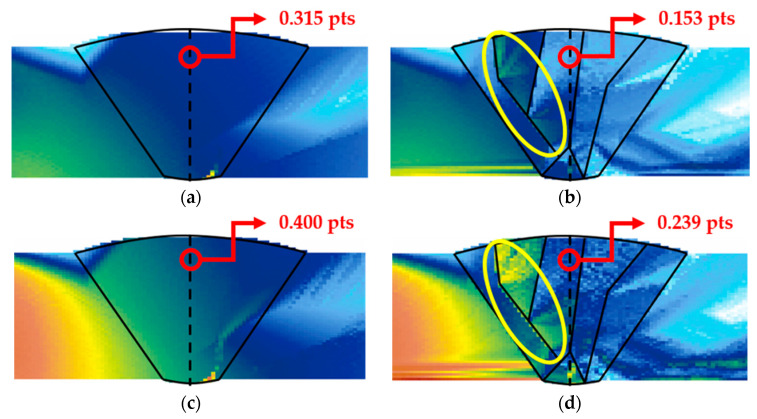
Beam computation results for transverse waves; at 7.5 MHz (**a**) isotropic and (**b**) classification into six domains; at 4 MHz (**c**) isotropic and (**d**) classification into six domains.

Figure 6 shows the beam computation results for the transverse waves. Figure 6a,c depict images of the isotropic welds, which indicate that the entire interior of the weld could be covered. In contrast, Figure 6b,d depict anisotropic welds, which exhibit refraction and scattering at weld interfaces, as well as the deflected propagation of the refracted ultrasonic beam at some interfaces. The overall intensity of the beam decreased due to beam distortion, and the propagation behavior of the beam intensity increased or decreased irregularly. To evaluate the difference in the beam attenuation depending on the frequency, the difference in the intensity of the beam was verified in a domain where significant beam attenuation was observed at a 1 skip distance. In this scenario, the pts unit used in the calculation is a default used in the CIVA software and represents the intensity of a signal. The intensity of the beam in the anisotropic weld decreased by approximately 40% compared to the isotropic weld at 4 MHz frequency, whereas it decreased by approximately 51% at a 7.5 MHz frequency. These results show that the transmittance improves with decreasing frequency, as described above.

**Figure 7:**
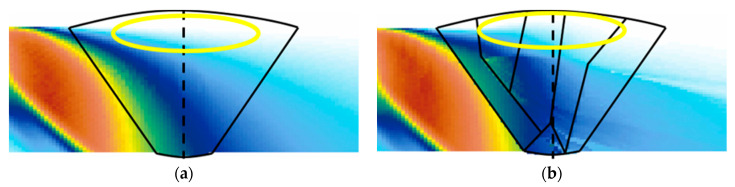
Beam computation results for longitudinal waves at 5 MHz (**a**) isotropic and (**b**) classification into six domains.

Figure 7 shows the beam computation results for the longitudinal waves. A comparison with the results presented in Figure 6 shows that less refraction and scattering occurred at the interfaces, unlike in the case of the transverse waves. This is attributed to the longer wavelength of the ultrasonic waves. Generally, longitudinal waves are approximately twice as fast as transverse waves, and their wavelength is approximately twice as long. Therefore, it can be inferred that longitudinal waves have a relatively smaller attenuation of the ultrasonic beam and higher transmittance compared to transverse waves. However, the inspection volume at the top of the weld could not be covered, because 0.5 skip was used, owing to the mode-converted signals. Furthermore, if the wavelength is long, it is likely that the flaw will be evaluated larger than it actually is. Consequently, longitudinal waves appear different from those of the transverse waves. For reliable and quantitative evaluation that covers the entire inspection volume, it is desirable to inspect the material using both transverse and longitudinal waves.

### 3.2. Beam Path Ray and Scan Plan

**Figure 8:**
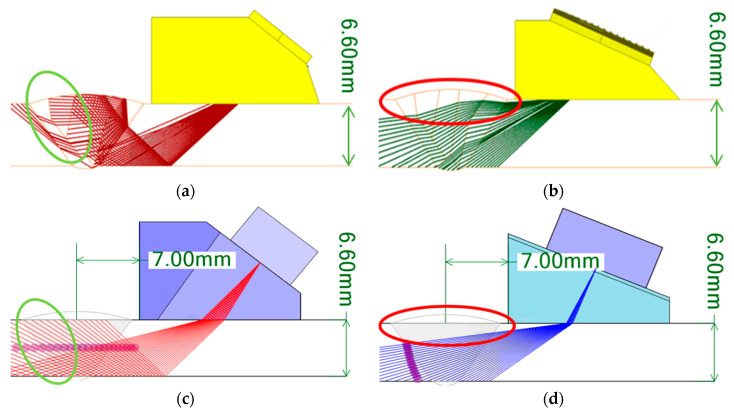
Scan plan for sectorial beam; classification into six domains (**a**) transverse wave scan plan and (**b**) longitudinal wave scan plan, isotropic (**c**) transverse wave scan plan and (**d**) longitudinal wave scan plan.

Figure 8 shows the scan plans prepared based on the beam computation results. The main parameters for the preparation of the scan plans were the index offset and angle of the sectorial beam. The index offset represents the distance from the center of the weld to the front part of the probe. Assuming that there is a bead in the weld, it is effective to place the probe closer to the weld and decrease the traveling distance of the beam for the inspection. As for the angle of the sectorial beam, it is necessary to set the angle for covering the inspection volume of the weld for the inspection. 

In this study, the beam path ray simulation was performed by varying the index offset and angle of the sectorial beam. Consequently, in the case of the transverse waves, as shown in Figure 8a,b, the scanning angle of the beam was set to 40°–75° to cover the inspection volume of the weld at a 1 skip distance, and the focal depth was set to 9.9 mm to focus on the center of the weld. In the case of the longitudinal waves, the scanning angle of the beam was set to 55°–85°, and the focal law was set to 18 mm using the half path to focus on the opposite weld interface. For both the longitudinal and transverse waves, the index offset was set to 7 mm. To verify the prepared scan plans, an isotropic weld was assumed according to the general scan plan preparation method, as shown in Figure 8c,d, and scan plans were prepared under the same conditions. In this scenario, Figure 8c,d show the inspection volume of the weld being covered. For the prepared scan plans, some of the inspection volume on the far side was not covered; however, this can be supplemented by bidirectional inspection. A reliable evaluation is also possible for asymmetrical welds through a bidirectional inspection.

## 4. Flaw Detection Sensitivity Analysis

### 4.1. Sensitivity Calibration

**Figure 9:**
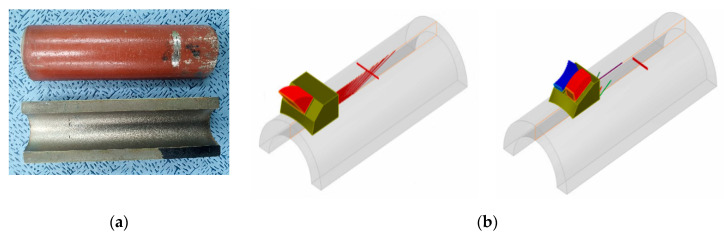
Specimen for sensitivity calibration and modeling; (**a**) sensitivity calibration block, (**b**) modeling for sensitivity calibration.

A-scan signals and S-scan images were used to analyze the PAUT results. In this case, setting the reference amplitude is important for the quantitative evaluation of flaws. Because the geometry and noise signals have been excluded in this simulation, only the A-scan signal reflected by the flaw is acquired. Therefore, it is important to determine the inspection method that acquires a high signal by comparing the sensitivity of the A-scan signal after setting the reference sensitivity for the three inspection conditions. To set the reference sensitivity for the flaw detection simulation, a sensitivity specimen with a diameter of 42.2 mm and thickness of 6.6 mm was modeled, as shown in Figure 9. Furthermore, a notch with a length of 6 mm and height of 1 mm was inserted into the outer and inner diameters of the specimen. The simulation was performed after setting the reference sensitivity, such that the transverse 60° and longitudinal 70° beams could be 100% reflected by the notches at 1 skip and 0.5 skip distances, respectively.

### 4.2. Flaw Detection

Flaws that occur during the manufacturing process and operation of thermal power plant boiler tubes include cracks, lack of fusion (LF), porosity, and slag. Among them, cracks and LF can cause the critical destruction of the structure as planar flaws [35,36]. To simulate planar flaws, the types of flaws were represented as rectangular flaws. Furthermore, to compare flaw detection sensitivity by the position of each flaw, the weld was divided into the upper, middle, and lower sections. Cracks were inserted at the center of the weld and LF on the left groove surface. Table 3 shows the characteristics of each flaw.

### 4.3. Simulation Results

#### 4.3.1. S-Wave Sectorial Scan

**Figure 10:**
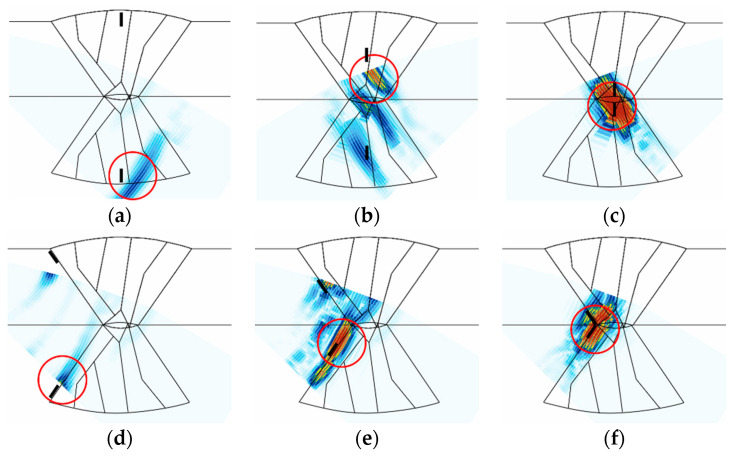
S-scan results of flaw detection simulation using transverse waves at a frequency of 4 MHz; (**a**) top crack, (**b**) middle crack, (**c**) bottom crack, (**d**) top LF, (**e**) middle LF, (**f**) bottom LF.

**Figure 11:**
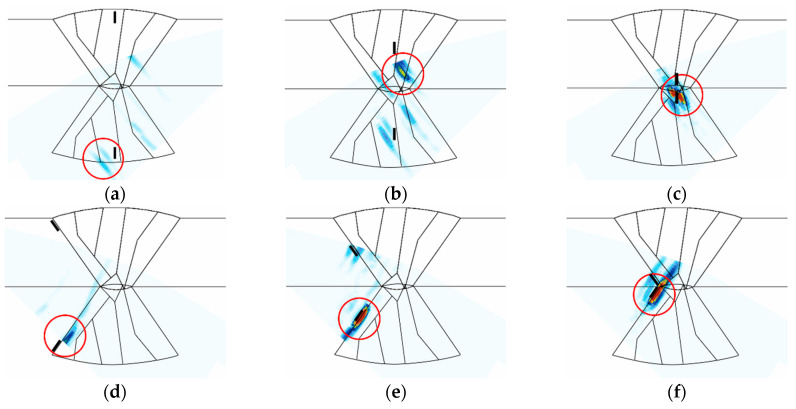
S-scan results of flaw detection simulation using transverse waves at a frequency of 7.5 MHz; (**a**) top crack, (**b**) middle crack, (**c**) bottom crack, (**d**) top LF, (**e**) middle LF, (**f**) bottom LF.

The flaw detection simulation was performed using transverse waves. The amplitudes of the A-scan signal for six types of flaws are summarized in Table 4. Figure 10 and Figure 11 depict the results for the 4 and 7.5 MHz frequencies, respectively, of the S-scan images for the higher A-scan signal between skew 90 and 270 in Table 4. In this scenario, skew 90 and 270 show the inspection results in the left and right directions, respectively. 

Table 4 indicates that the amplitudes for the top crack did not exceed 20% of the reference sensitivity at 7.5 MHz, as they achieved 7.16% and 8.46%. However, at 4 MHz frequency, the amplitudes were 27.78% and 23.37%, confirming higher signals compared to the scenario at 7.5 MHz frequency. Overall, the amplitudes of the A-scan signal were higher at 4 MHz frequency. This is attributed to the lower frequency having a higher transmittance of the ultrasonic beam in the beam computation result. However, a comparison between Figure 10c and Figure 11c shows that the flaw was evaluated to be larger than actual size when flaw detection was performed using the lower frequency, owing to the spread of the beam. Figure 10b and Figure 11b show that there are other signals in high amplitudes as well as the actual flaw signals. These signals are assumed to be scattering and diffraction signals of the beam, and these unrelated signals can make it difficult to evaluate the position and size of the flaw. For the LF located on the groove surface of the weld, high A-scan signals can be observed at skew 90 (left groove surface) from the results presented in Table 4, as the ultrasonic beam vertically reflected off the flaw without passing through the weld with 1 skip. A-scan signals higher than the reference sensitivity were also observed at skew 270; however, the signals were relatively low due to the distortion of the beam as it passed through the weld. In the case of top LF, amplitude is almost zero, such that the flaw cannot be detected. These results indicate that the 4 MHz frequency, which represents a relatively low frequency, is suitable for flaws that exist inside the weld and side wall.

#### 4.3.2. L-Wave Sectorial Scan

**Figure 12:**
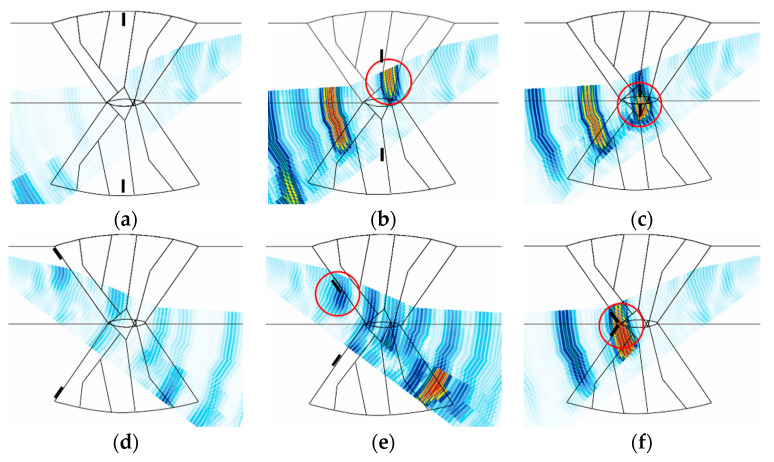
S-scan results of flaw detection simulation using longitudinal waves at a frequency of 5 MHz; (**a**) top crack, (**b**) middle crack, (**c**) bottom crack, (**d**) top LF, (**e**) middle LF, (**f**) bottom LF.

Table 5 shows the amplitude of the A-scan signal, and Figure 12 shows the S-scan images of the flaw detection simulation. In Figure 12, only the results for the high A-scan signals in the bidirectional inspection are shown. In the overall simulation results, mode-converted signals can be identified behind the flaw signals, owing to the mode conversion that occurred because of the reflection of the longitudinal waves. These unrelated signals can make it difficult to evaluate the position and size of the flaw. However, in the case of the cracks located inside the weld, mode-converted signals, which are unrelated signals, were located outside a 0.5 skip distance, which was the region of interest. Therefore, relatively clean images of the flaw could be obtained in the region of interest in comparison to the S-scan image results of Figure 10b and Figure 11b. 

However, the top crack could not be detected. These results are similar to those expected when the scan plans were prepared. Both the middle and bottom cracks exhibited excellent A-scan signals. As for the LF on the groove surface of the weld, the top LF could not be detected in the same manner. The middle LF was detected with low signals. This may be because the signals were not properly received from the flaw, as the longitudinal waves used only 0.5 skip and the traveling direction of the beam was almost parallel to the slope of the flaw. Furthermore, it was difficult to identify flaw signals because of the mode-converted signals behind them. Unlike in the case of the transverse waves, the bottom LF exhibited a higher A-scan signal because the ultrasonic beam is vertically reflected off the flaw at skew 270.

## 5. Experimental Setup

**Figure 13:**
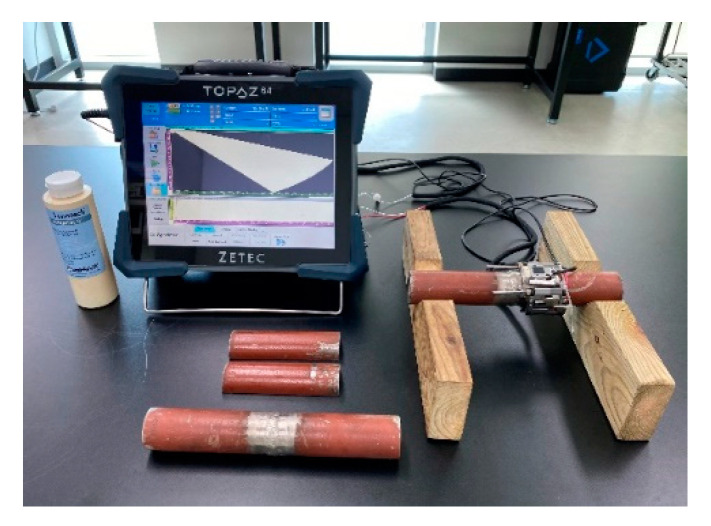
Experimental setup of specimens and equipment.

**Figure 14:**
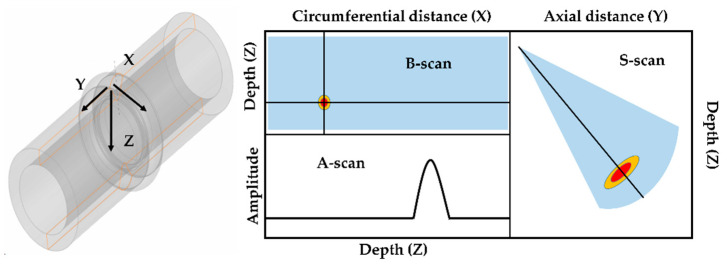
Description of A-, B-, and S-scans.

We carried out an experiment to verify the validity of the simulation results. The specimens, probes, and inspection method used in the experiment were identical to those in the simulation. Figure 13 shows the experimental setup of specimens and equipment. The PAUT detector used for the inspection was Zetec’s TOPAZ 64. For each of the experiments, basic calibration was performed for the flaw detector and probe. Thereafter, the reference amplitude was set using the sensitivity calibration block. After the calibration was completed, the experiment was carried out after installing the encoder for the flat flaw of 6mm length and 1mm height under the same conditions as the simulation. When the PAUT experiment was carried out with the encoder installed, the S-scan, B-scan, and A-scan could be checked by default. Information about each scan is shown in Figure 14.

**Figure 15:**
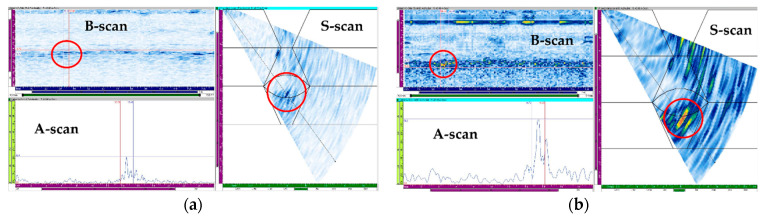
Experimental sectorial scan results for top crack with (**a**) 7.5 MHz transverse wave and (**b**) 4 MHz transverse wave.

**Figure 16:**
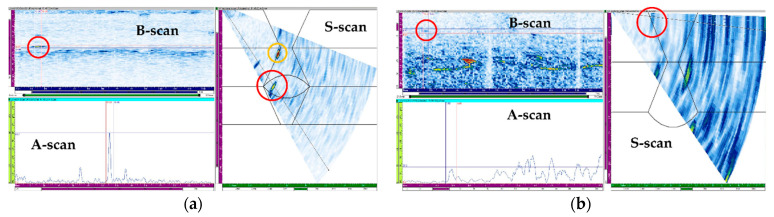
Experimental sectorial scan results for the top LF with (**a**) 7.5 MHz transverse wave and (**b**) 5 MHz longitudinal wave.

**Figure 17:**
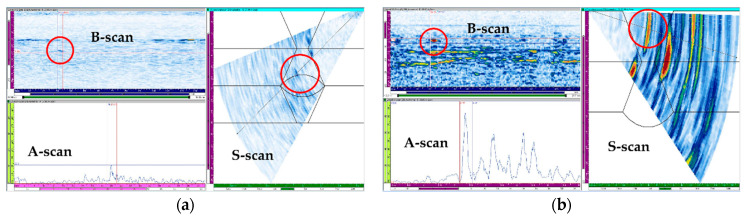
Experimental sectorial scan results for the middle crack with (**a**) 7.5 MHz transverse wave and (**b**) 5 MHz longitudinal wave.

The flaw detection results of simulation and experiment are shown in Table 6. The simulation and experimental results showed that all flaws were detected when three scan plans were used in parallel. Simulation and experimental results for six types of flaw showed that only the 4 MHz frequency could generate valid A-scan signals for the top crack. Top LF could be detected when transverse waves were used but were not detected when the longitudinal wave was used. For the middle crack, detection was possible in all scan plans. However, when evaluating flaws using transverse waves, it can be difficult to evaluate flaws due to scattering and diffraction signals. Therefore, it was suitable for flaw evaluation to perform the inspection using the longitudinal wave. Figure 15, Figure 16 and Figure 17 show the experimental results for the three cases mentioned above.

For the top crack, inspection was performed using the probes for the transverse waves at frequencies of 4 and 7.5 MHz, and the results are shown in Figure 15. Although the scan plans were prepared based on 1 skip inspection for the transverse waves, high A-scan signals were obtained in the 1.5 skip region, as shown in Figure 15. This may be due to the distortion of the ultrasonic wave, which occurred owing to the anisotropy and inhomogeneity of the austenitic steel weld, and the flaw signals were detected at a different position compared to the actual flaw signals. The results of the inspection using the 7.5 MHz probe indicated a flaw signal higher than ~20%; however, it was difficult to identify it as a flaw signal, as geometry signals of similar amplitudes occurred continuously at the same depth, according to the B-scan. With the 4 MHz probe, the B-scan demonstrated geometry signals at the same depth; however, it exhibited a higher signal around the flaw, which was identified as a flaw signal. These results were similar to the simulation results.

For the top LF, inspection was performed using the probe for the transverse and longitudinal waves at frequencies of 7.5 and 5 MHz, respectively; the results are shown in Figure 16. The results of the inspection using the 5 MHz probe, a signal judged to be LF was detected at the upper left. However, it was determined that the amplitude of the signal reflected from the flaw was less than 20%, making it difficult to detect the flaw. On the other hand, using a 7.5 MHz probe made it possible to detect the flaw with a relatively high A-scan signal. In Figure 16a, a high A-scan signal was detected not only from the flaw location but also from the bottom of the weld. This A-scan signal was determined to be a signal from the bead of the bottom.

For the middle crack, the inspection was performed using the probe for the transverse and longitudinal waves at frequencies of 7.5 and 5 MHz, respectively; the results are shown in Figure 17. The results of the inspection using the 7.5 MHz probe could obtain a flaw signal of approximately 30%; however, there were limitations in identifying the position and type of flaw due to the low signal amplitude, and the scattering and refraction of the ultrasonic waves. The results of the inspection using the 5 MHz probe showed a flaw signal above 80%, and the position of the flaw could be accurately determined. Although the longitudinal waves were highly likely to evaluate a flaw to be larger because of the spread of the beam, they exhibited higher detection sensitivity than the transverse waves for the anisotropic welds.

## 6. Conclusions

This study was conducted to verify the reliability of the phased array ultrasonic testing (PAUT) technique and improve the probability of detection (POD) for the inspection of anisotropic and inhomogeneous welds, such as austenitic steel welds.

To achieve this, weld modeling was performed by reflecting the metal crystal structure of the weld, and the optimal scan plan was derived from the propagation behavior of PA ultrasonic beams in the welds and beam ray path simulation. The derived scan plan was prepared by taking into account the wave mode and frequency, and inspection was performed on both sides. The flaw detection sensitivity was analyzed by simulation under these inspection conditions, and we found that all flaws could be detected. It was also confirmed that the simulation technique derived from verification through the experiment showed the same result as the experiment. However, flaw positioning or sizing performance could not be accurately evaluated. Accurately evaluating the location and size of defects (sizing performance and positioning) is very important for inspection. However, as mentioned in the introduction to this paper, it is difficult to detect flaws in a material that exhibits anisotropy, such as austenitic steel in actual inspection and attenuates significantly due to coarse grains. Evaluating the location and size of the flaw is also an important issue, but detection of the flaw must precede it, and it was judged most important. This is because it is impossible to evaluate its size and location if the defect detection itself is difficult. The S-scan images used for the evaluation of the positioning or sizing performance were created using the collected A-scan signals and the beam path of the ultrasonic waves. The flaw signals occurred at positions different to the actual flaw positions. This is because the distortion of the beam caused by the anisotropy of the weld made it difficult to predict the accurate beam path of the ultrasonic waves. Further research on imaging techniques is required for a more reliable evaluation. 

In the future, research and additional experiments on model-assisted POD that consider a larger number of parameters will be conducted using such simulation modeling techniques to verify the reliability of PAUT for finding flaws in welds.

## Figures and Tables

**Table 1:** Calculation time according to the modeling method.

Anisotropic Model	Ogilvy	3 Domains	6 Domains	12 Domains	24 Domains
Total time	30 min	30 min	50 min	180 min	300 min

**Table 2:** Specifications of probe.

Wave Type	Longitudinal	Transverse	
Center Frequency	5 MHz	4 MHz	7.5 MHz
Number of Elements	16 × 2 (TR)	16	
Element Pitch	0.75 mm	0.5 mm	
Total Aperture	12 mm	8 mm	
Elevation	5 mm	10 mm	

**Table 3:** Flaw dimensions of simulation.

Shape	Rectangular		
Length	6 mm		
Height	1 mm		
Depth	0 mm	3 mm	6 mm
Crack	** 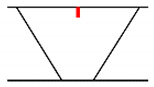 **	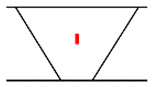	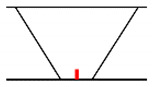
Lack of Fusion (LF)	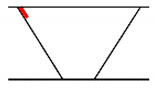	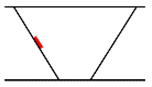	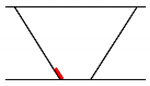

**Table 4:** Amplitudes of A-scan for six types of flaws using transverse wave.

Flaw Type	Frequency (MHz)	Amplitude (%)	
		Skew 90	Skew 270
Top Crack	4	27.78	23.37
	7.5	7.16	8.46
Middle Crack	4	70.76	100.34
	7.5	35.56	62.38
Bottom Crack	4	214.67	374.52
	7.5	100.88	229.68
Top LF	4	59.51	27.86
	7.5	47.1	Not Detected
Middle LF	4	198.19	96.08
	7.5	225.4	33.06
Bottom LF	4	219.36	191.27
	7.5	236.42	40.64

**Table 5:** A-scan results of flaw detection simulation using longitudinal waves.

Flaw Type	Frequency (MHz)	Amplitude (%)	
		Skew 90	Skew 270
Top Crack	5	Not detected	Not detected
Middle Crack		62.86	101.1
Bottom Crack		95.43	109.16
Top LF		Not detected	Not detected
Middle LF		33.75	26.23
Bottom LF		98.85	193.98

**Table 6:** Results of flaw detect simulation and experiment.

Flaw Type	Frequency (MHz)	Detection	
		Simulation	Experiment
Top Crack	4	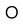	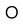
	7.5	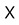	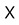
	5	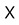	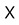
Top LF	4	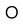	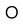
	7.5	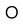	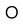
	5	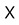	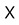
Others	4	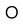	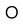
	7.5	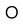	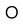
	5	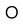	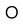

## Data Availability

Data sharing not applicable.
